# Community norms for the Eating Disorder Examination Questionnaire (EDE-Q) among gender-expansive populations

**DOI:** 10.1186/s40337-020-00352-x

**Published:** 2020-12-08

**Authors:** Jason M. Nagata, Emilio J. Compte, Chloe J. Cattle, Annesa Flentje, Matthew R. Capriotti, Micah E. Lubensky, Stuart B. Murray, Juno Obedin-Maliver, Mitchell R. Lunn

**Affiliations:** 1 Department of Pediatrics, University of California, San Francisco, 550 16th Street, 4th Floor, Box 0110, San Francisco, CA 94158 USA; 2 Eating Behavior Research Center School of Psychology, Universidad Adolfo Ibáñez, Santiago, Chile; 3 Research Department, Comenzar de Nuevo Treatment Center, Monterrey, Mexico; 4 Department of Community Health Systems, University of California, San Francisco, San Francisco, CA USA; 5 Alliance Health Project, Department of Psychiatry and Behavioral Sciences, University of California, San Francisco, San Francisco, CA USA; 6 The PRIDE Study/PRIDEnet, Stanford University School of Medicine, Stanford, CA USA; 7 Department of Psychology, San José State University, San Jose, CA USA; 8 Department of Psychiatry and Behavioral Sciences, University of Southern California, Los Angeles, CA USA; 9 Department of Obstetrics and Gynecology, Stanford University School of Medicine, Stanford, CA USA; 10 Department of Epidemiology and Population Health, Stanford University School of Medicine, Stanford, CA USA; 11 Division of Nephrology, Department of Medicine, Stanford University School of Medicine, Stanford, CA USA

**Keywords:** Gender-expansive, Non-binary, Genderqueer, Gender fluid, Eating disorder, Disordered eating

## Abstract

**Purpose:**

Gender-expansive individuals (i.e., those who identify outside of the binary system of man or woman) are a marginalized group that faces discrimination and have a high burden of mental health problems, but there is a paucity of research on eating disorders in this population. This study aimed to describe the community norms for the Eating Disorder Examination Questionnaire (EDE-Q) in gender-expansive populations.

**Methods:**

The participants were 988 gender-expansive individuals (defined as neither exclusively cisgender nor binary transgender) from The PRIDE study, an existing longitudinal cohort study of health outcomes in sexual and gender minority people.

**Results:**

We present the mean scores, standard deviations, and percentile ranks for the Global score and four subscale scores of the EDE-Q in this group as a whole and stratified by sex assigned at birth. Gender-expansive individuals reported any occurrence (≥1/28 days) of dietary restraint (23.0%), objective binge episodes (12.9%), excessive exercise (7.4%), self-induced vomiting (1.4%), or laxative misuse (1.2%). We found no statistically significant differences by sex assigned at birth. Compared to a prior study of transgender men and women, there were no significant differences in eating attitudes or disordered eating behaviors noted between gender-expansive individuals and transgender men. Transgender women reported higher Restraint and Shape Concern subscale scores compared to gender-expansive individuals. Compared to a prior study of presumed cisgender men 18–26 years, our age-matched gender-expansive sample had higher Eating, Weight, and Shape Concern subscales and Global Score, but reported a lower frequency of objective binge episodes and excessive exercise. Compared to a prior study of presumed cisgender women 18–25 years, our age-matched gender-expansive sample had a higher Shape Concern subscale score, a lower Restraint subscale score, and lower frequencies of self-induced vomiting, laxative misuse, and excessive exercise.

**Conclusions:**

Gender-expansive individuals reported lower Restraint and Shape Concern scores than transgender women; higher Eating, Weight, and Shape Concern scores than presumed cisgender men; and lower Restraint but higher Shape Concern scores than presumed cisgender women. These norms can help clinicians in treating this population and interpreting the EDE-Q scores of their gender-expansive patients.

## Plain English summary

Gender-expansive describes gender identities that do not fit within the binary gender identity system. We asked gender-expansive participants in The PRIDE Study to fill out a widely used survey about eating disorder attitudes and behaviors. Nearly one quarter of gender-expansive individuals reported dietary restraint in the past 28 days. Thirteen percent of gender-expansive individuals reported binge eating in the past 28 days. Eating disorder attitudes and behaviors in gender-expansive individuals did not differ significantly based on sex assignment at birth. Gender-expansive people had similar rates of eating disorder symptoms as transgender men and reported lower restraint symptoms and concerns about shape than transgender women. These norms can help clinicians and researchers in interpreting eating disorder attitudes and behaviors in gender-expansive populations.

## Introduction

Gender-expansive describes a spectrum of gender identities that do not fit within the binary gender identity system (i.e., man or woman). This includes those who identify with no particular gender, with some combination of man and woman, and those whose gender identity shifts over time. Although the terminology of the community is continuously evolving, some identities that fall within this spectrum include genderqueer (a term that is often used synonymously with gender non-binary), pangender (i.e., identification with multiple or all genders), and gender fluid (i.e., a gender identity that shifts over time). Research on health outcomes in this minority population is limited, and, when research is presented, it generally conflates all gender-expansive identities and experiences with transgender (when an individual’s gender identity differs from the sex assigned to them at birth) identities and experiences, even though the terms are not synonymous [1]. Specifically, only some transgender individuals identify as gender-expansive or non-binary, and not all people who identify as gender-expansive or non-binary also identify as transgender. Thus, conflating these distinct groups creates the potential for inaccurate findings relating to distinct groups, thus marginalizing distinct identities. For instance, accumulating evidence suggests that gender-expansive individuals face increased levels of overall psychological distress, have less social support, experience more cyberbullying, and have poorer mental health outcomes compared to transgender and cisgender people [2–4]. As such, there is a pressing need to characterize the specific risk factors and mental health outcomes of gender-expansive people.

To date, the majority of eating disorder (ED) research has focused on cisgender women. The limited research on eating pathology in gender minority people often combines sexual and gender minority (SGM) people (i.e., individuals who identify as lesbian, gay, bisexual, transgender, queer, asexual, and/or intersex, and those whose sexual orientation and/or gender identity falls outside of binary constructs) [5] into one group for analyses even though sexual orientation and gender identity are distinct [6]. Even the literature that focuses on gender minority populations (i.e.*,* those whose gender identities do not align with that commonly associated with their sex assigned at birth, e.g., transgender, genderqueer, agender, and/or gender non-binary) usually collapses this diverse group of identities into one, fails to separate binary transgender from gender-expansive individuals, or excludes gender-expansive individuals. With this limitation in mind, current research on eating pathology in SGM people suggests that these individuals have higher rates of ED self-diagnosis [7], engage in more compensatory behaviors (e.g., use of diet pills and laxatives), and have more severe ED symptomatology [7, 8]. Specifically, it has been found that SGM people have higher Eating Disorder Examination Questionnaire (EDE-Q) scores at admission to treatment compared to cisgender heterosexual individuals [9]. Furthermore, it has been suggested that, in SGM people, disordered eating behaviors may be associated with other mental health outcomes and a decreased quality of life [10]. The scant literature that compares disordered eating across individuals with different minority gender identities suggests that there may be important differences in ED prevalence with gender-expansive individuals who were assigned a female sex at birth having an increased risk of an ED relative to transgender men or women [6] and gender-expansive youth reporting higher dietary restraint than binary transgender youth [11]. Since the vulnerability of gender-expansive individuals to eating pathology is poorly understood, it is crucial that we characterize the community norms in this marginalized group and seek to understand how it may differ from their exclusively cisgender or binary transgender peers.

There are multiple factors that may contribute to the susceptibility of gender-expansive individuals to eating disorders. Gender identity and body image are linked. Ålgars et al. [12] found that individuals with gender dysphoria also reported more dissatisfaction with their bodies compared to matched controls without gender dysphoria and, in women, these individuals had increased signs of disordered eating. This may, in part, be accounted for by the cis-sexist (prejudice or discrimination based on the assumption that people are cisgender) and anti-transgender discrimination that this population faces. For example, Tabaac et al. [13] found that discrimination was negatively correlated with body appreciation, and studies have found that transgender individuals who are more visually gender-nonconforming experience more public discrimination. Additionally, Himmelstein et al. [14] found that over half of their sample of SGM people experienced weight-based violence from peers or family members; these experiences have been found to be associated with maladaptive eating behaviors. More broadly, the Gender Minority Stress Model has been used to describe unique stressors faced by transgender and non-binary individuals that contribute to negative health outcomes [15, 16]. Specifically, this population experiences high levels of gender-based victimization and discrimination and a greater amount of stressful life events as a result of their gender expression, which are each associated with negative mental health sequelae [15]. Johnson et al. [17] have described invalidation (“the refusal to accept someone’s identity as ‘real’ or ‘true’”) as a unique form of minority stress that impacts gender-expansive individuals and is a contributing factor to significant psychological distress. Bell et al. [18] found that perceived stigma was indirectly associated with eating disorder proneness that was mediated by self-compassion among transgender and gender-nonconforming adults.

The Eating Disorder Examination Questionnaire (EDE-Q) is a widely used assessment of ED attitudes and behaviors. Although limited literature suggests that gender-expansive individuals may be at an increased risk of EDs, there is still a paucity of empirical data on this specific population [6, 19]. To address this gap in the literature, the goal of the present study was to characterize the community norms for the EDE-Q among gender-expansive individuals. A second objective was to compare these EDE-Q norms of gender-expansive individuals to previously published norms of transgender men [20], transgender women [20], presumed cisgender men [21], and presumed cisgender women [22]. This is crucial to understanding how eating pathology manifests in a population that is often merged with or excluded from other gender minority groups, which may obscure potential key differences.

## Methods

### Study design and population

The Population Research in Identity and Disparities for Equality (PRIDE) Study is a longitudinal national cohort study of adults who identify as a sexual and/or gender minority (SGM), including, but not limited to, lesbian, gay, bisexual, transgender, and queer individuals. Specific inclusion criteria included: age ≥ 18 years, self-identification as a sexual and/or gender minority, living in the U.S. or its territories, and the ability to read and respond to a questionnaire written in English. Study participants were recruited via digital advertisements and communications, in-person outreach, and distribution of promotional materials. These recruitment efforts have been led by PRIDEnet, a national community engagement network created to engage SGM communities in all aspects of The PRIDE Study. Full demographics of the cohort population and description of the technology that supports The PRIDE Study have been reported previously [23]. For the present study, data were collected by inviting all participants of The PRIDE study to complete a questionnaire entitled “Eating and Body Image” between April 2018 to August 2018. The study was approved by the Stanford University and University of California, San Francisco Institutional Review Boards as well as The PRIDE Study’s Participant and Research Advisory Committees. Written informed consent was obtained of all participants.

Participants were asked about their gender identity (“What is your current gender identity?”) and were able to choose more than one option and/or write in their identity if it was not provided in the preset categorical answer choices. They were also asked to identify the sex assigned to them at birth (“What sex were you assigned at birth on your original birth certificate?”). For this particular study, we excluded those who were classified as a cisgender man, cisgender woman, transgender man, or transgender woman (see Appendix for definitions) [20, 24, 25]. Specifically, this analysis included those who selected “genderqueer,” multiple gender identities, and/or those who selected “another gender identity” and/or provided a write-in (such as non-binary, nonconforming, genderfluid, agender, and bigender). Of the 4672 participants from The PRIDE Study who completed the questionnaire, 1120 could be classified as gender-expansive by the reported criteria. However, 132 participants had missing values on EDE-Q items (average of 55.6% items missing) and were excluded from the analyses; thus, 988 participants were included in the current study. From the final sample, 1.5% (*n* = 15) did not report data on sex assigned to them at birth and were not included in the sex-based comparisons. EDE-Q norms of included gender-expansive individuals were then compared to norms of transgender men (*n* = 312) and transgender women (*n* = 172) previously reported from The PRIDE Study [20]. In addition, we selected previously published norms of presumed cisgender men [21] and women [22] as comparison groups as they were samples that most closely matched The PRIDE Study’s samples (e.g., U.S.-based, non-clinical, community samples of adults). However, these previously published samples were of young adults; thus, we analyzed a subset of age-matched gender-expansive individuals 18–26 years (*n* = 483) for comparisons with presumed cisgender men 18–26 years [21] and age-matched gender-expansive individuals 18–25 years (*n* = 434) for comparisons with presumed cisgender women 18–25 years [22]. Although a prior study [20] used an Australian sample of presumed cisgender women 18–42 years as a comparison group [26], we chose the US sample of presumed cisgender women 18–25 years as the comparison group [22] given that our age-matched comparison group of gender-expansive individuals was sufficiently large for comparisons (*n* = 434). In this way, the two presumed cisgender comparison groups were similar in young adult age range [21, 22], and both of the the two comparison groups were US-based samples.

### Measures

The EDE-Q is a widely used self-report questionnaire that assesses a range of disordered eating attitudes and behaviors over the previous 28 days [27]. This 28-item measure uses a forced-choice 7-point rating scale (0–6) for each item, with higher scores reflecting greater symptom occurrence. The items of the measure assess different aspects of ED pathology and enable the calculation of four subscale scores: Restraint (5 items), Eating Concern (EC, 5 items), Shape Concern (SC, 8 items), and Weight Concern (WC, 5 items). The global score is calculated as a weighted average of the subscale scores. In this study, internal consistency was .95 for the global score; .83 for Restraint; .84 for EC; .90 for SC; and .85 for WC.

The frequency of specific behaviors was assessed by the EDE-Q in terms of number of occurrences within the previous 28 days and defined using cutoffs from previously published EDE-Q norms studies [20–22, 24, 25, 28–31] in order to allow for comparisons across studies. For all behaviors assessed, any occurrence was defined as ≥1 episode in the previous 28 days [21, 22]. For objective binge episodes, self-induced vomiting, and laxative misuse, a regular occurrence was defined as ≥4 episodes in the past 28 days [21, 22], which would average to at least one episode per week and is consistent with the current Diagnostic and Statistical Manual, 5th Edition (DSM-5) criteria for bulimia nervosa and binge eating disorder [32]. Regular occurrence of dietary restraint referred to going for long periods of time (8 h) without eating anything in order to influence shape or weight for ≥13 days over the past 28 days as has been previously defined [21, 22, 26]. The choice of the cut-off for dietary restraint was based on ≥3 on the 0–6 scale of the EDE-Q item 2, corresponding to an average of > 3 days per week [22]. A rating of 4 on items/subscales of the EDE-Q has been used as a cut-off for clinical severity [21], so the selection of the cut-off corresponding to a rating of ≥3 allowed for inclusion of a somewhat lower frequency given that the fasting behavior represented an extreme form of dietary restraint [21, 26]. Regular excessive exercise was defined as exercising in a driven or compulsive way as a means of controlling weight, shape or amount of fat, or burning off calories for ≥20 days over the past 28 days [21, 22]. The choice of the cut-off for driven or compulsive exercise was based on an average of ≥5 days per week, and this higher threshold was selected in order to enhance the likelihood that the cut-off reflects clinical severity, given that the item may not clearly distinguish between pathological versus adaptive forms of exercise [21, 22].

Demographic information was also collected from the participants. Specifically, they were asked to report their age, race, ethnicity, education, height, and weight. Body mass index (BMI) was calculated from self-reported height and weight using the standard formula (BMI = weight/height^2^), with weight in kilograms and height in meters. Additionally, participants were asked: “Has a mental health professional or physician ever told you that you have an eating disorder such as anorexia nervosa, bulimia nervosa, or binge eating disorder?” They were asked to report the ED(s) with which they had been diagnosed, and the options included anorexia nervosa, bulimia nervosa, binge eating disorder, or other/not specified.

### Data analysis

SPSS 20.0 was used for all statistical analyses. Associations between participants’ BMI and scores on the EDE-Q subscales and global measure were assessed through the Pearson product-moment correlation coefficient. We calculated norms for all individuals in The PRIDE Study who were classified as gender-expansive, combining those assigned male sex at birth (AMAB) and those assigned female sex at birth (AFAB), as well as participants who did not report their sex assigned at birth in our initial analysis. For sensitivity analyses, we performed separate calculations of the norms for gender-expansive individuals AMAB (*n =* 135) and those AFAB (*n* = 838) in order to determine differences based on sex assigned at birth. Student t-test for independent samples and Chi-square analyses were conducted to assess differences between AMAB and AFAB participants, and between gender-expansive and previous published norms on transgender men and women, as well as previous norms on presumably cisgender men and women. Finally, a two-tailed threshold of *p* < .005 was used after Bonferroni’s correction.

## Results

A total of 988 gender-expansive individuals were included in this study. The mean age was 29.5 years (*SD =* 9.2, range 18–71), the mean BMI was 28.5 kg/m^2^ (*SD* = 8.3, range 14.5–66.9), and 63% had completed a college degree or higher. In total, 79.3% of the gender-expansive individuals identified as White, 2.8% as Asian, 1.1% as Black, 0.3% as Native American/American Indian, 11.3% as multiracial, 3.9% as another race, and 1.3% did not report their race/ethnicity. Additionally, a total of 5.7% of participants identified as Hispanic, Latino, or of Spanish origin.

The mean EDE-Q subscales and global scores, along with standard deviations and percentile ranks, are shown in Table 1. Overall, 13.8% of participants reported being told by a mental health provider or physician that they had an eating disorder, including anorexia nervosa (6.1%), bulimia nervosa (2.5%), binge eating disorder (2.1%), or other/not specified (3.6%). BMI showed significant positive weak to moderate correlations with all EDE-Q subscales (BMI vs. Restraint: *r* = .16, *p* < .001; BMI vs. EC: *r* = .27, *p* < .001; BMI vs. WC: *r* = .40, *p* < .001; BMI vs. SC: *r* = .33, *p* < .001; BMI vs. EDE-Q GS: *r* = .33, *p* < .001).

**Table 1:** Distribution of means, standard deviations, and percentile ranks for Eating Disorder Examination Questionnaire (EDE-Q) Global and subscale scores among gender-expansive individuals from The PRIDE Study (*N* = 988)

	EDE-Q Restraint	EDE-Q Eating Concern	EDE-Q Weight Concern	EDE-Q Shape Concern	EDE-Q Global
M (SD)	1.24 (1.49)	1.02 (1.26)	2.18 (1.63)	2.58 (1.65)	1.76 (1.33)
Range	0–6.00	0–6.00	0–6.00	0–6.00	0–5.95
Percentile rank					
5	0.0	0.0	0.0	0.3	0.2
10	0.0	0.0	0.2	0.5	0.3
15	0.0	0.0	0.4	0.8	0.4
20	0.0	0.0	0.6	1.0	0.5
25	0.0	0.0	0.8	1.1	0.7
30	0.0	0.2	1.0	1.4	0.8
35	0.0	0.2	1.2	1.6	0.9
40	0.2	0.2	1.4	1.9	1.1
45	0.4	0.4	1.6	2.1	1.3
50	0.6	0.4	1.9	2.4	1.4
55	0.8	0.6	2.2	2.8	1.6
60	1.2	0.8	2.4	2.9	1.9
65	1.4	1.0	2.8	3.3	2.1
70	1.8	1.2	3.0	3.5	2.3
75	2.2	1.6	3.4	3.9	2.6
80	2.6	2.0	3.8	4.3	2.9
85	3.0	2.4	4.2	4.5	3.3
90	3.6	2.8	4.6	5.0	3.7
95	4.3	3.8	5.2	5.5	4.5
99	5.8	5.2	6.0	6.0	5.5

*M* Mean, *SD* Standard deviation

Any occurrence and regular occurrences of key ED behavioral features and compensatory behaviors are presented in Table 2. Approximately 13% of the sample reported at least one episode of objective binge eating during the previous 28 days, and 23% reported at least one episode of dietary restriction in the previous 28 days. Also, 1.4 and 1.2% of the participants reported purging methods such as self-induced vomiting and laxative misuse, respectively, and a little more than 7% reported any occurrence of excessive exercise in the previous 28 days.

**Table 2:** Proportion of gender-expansive individuals engaging in disordered eating behaviors among 988 participants in The PRIDE Study

Disordered eating behavior	Any occurrence		Regular occurrence	
	%	n	%	n
Dietary restraint	23	227	7.4	73
Objective binge episodes	12.9	127	6.8	67
Self-induced vomiting	1.4	14	0.9	9
Laxative misuse	1.2	12	0.8	8
Excessive exercise	7.4	73	1.2	12

Any occurrence was defined as ≥1 episode in the past 28 days. Regular occurrence of dietary restraint was defined as going for long periods of time (≥8 h) without eating anything to influence shape or weight for ≥13 days over the past 28 days. Regular occurrence of excessive exercise was defined as exercising in a driven or compulsive way as a means of controlling weight, shape or amount of fat, or burning off calories for ≥20 days over the past 28 days. For all other behaviors (objective binge episodes, self-induced vomiting, and laxative misuse), regular occurrence was defined as ≥4 occurrences over the past 28 days. Definitions for any and regular occurrence are consistent with previously published EDE-Q norms studies [20–22, 24, 25, 28–30]

Table 3 shows mean EDE-Q subscales and global scores, along with standard deviations and percentile ranks, for AMAB and AFAB gender-expansive participants. No significant differences were observed between AMAB and AFAB gender-expansive participants for the Restraint (*t =* − 1.37, *p = .*170), EC (*t =* 1.60, *p =* .109), WC (*t =* 0.18, *p =* .854), and SC (*t = −* 0.43, *p =* .666) subscales and for the EDE-Q GS (*t =* − 0.08, *p =* .934). In addition, occurrences of key ED behavioral features and compensatory behaviors in AMAB and AFAB gender-expansive participants are presented in Table 4. No significant differences were observed between AMAB and AFAB gender-expansive participants for any and regular occurrences of key ED behaviors (see Table 4).

**Table 3:** Distribution of means, standard deviations, and percentile ranks for the Eating Disorder Examination Questionnaire (EDE-Q) Global and subscale scores among gender-expansive individuals assigned a male sex at birth (*N* = 135) and gender-expansive individuals assigned a female sex at birth (*N* = 838) from the PRIDE Study

	Gender-expansive assigned male at birth (*N* = 135)					Gender-expansive assigned female at birth (*N* = 838)				
	EDE-Q Restraint	EDE-Q Eating Concern	EDE-Q Weight Concern	EDE-Q Shape Concern	EDE-Q Global	EDE-Q Restraint	EDE-Q Eating Concern	EDE-Q Weight Concern	EDE-Q Shape Concern	EDE-Q Global
M (SD)	1.38 (1.61)	0.84 (1.21)	2.14 (1.55)	2.62 (1.62)	1.75 (1.31)	1.20 (1.45)	1.03 (1.25)	2.17 (1.64)	2.55 (1.64)	1.74 (1.32)
Range	0–6.00	0–5.20	0–6.00	0–6.00	0–5.76	0–6.00	0–6.0	0–6.0	0–6.0	0–5.95
Percentile rank										
5	0.0	0.0	0.0	0.1	0.1	0.0	0.0	0.0	0.3	0.2
10	0.0	0.0	0.2	0.5	0.3	0.0	0.0	0.2	0.5	0.3
15	0.0	0.0	0.6	0.8	0.4	0.0	0.0	0.4	0.8	0.4
20	0.0	0.0	0.8	0.9	0.6	0.0	0.0	0.6	1.0	0.5
25	0.0	0.0	0.8	1.1	0.7	0.0	0.0	0.8	1.1	0.7
30	0.0	0.0	1.0	1.4	0.8	0.0	0.2	1.0	1.4	0.8
35	0.2	0.2	1.1	1.8	1.0	0.0	0.2	1.2	1.6	0.9
40	0.4	0.2	1.5	2.0	1.2	0.2	0.2	1.4	1.9	1.1
45	0.6	0.2	1.6	2.4	1.3	0.4	0.4	1.6	2.1	1.3
50	0.8	0.4	2.0	2.8	1.5	0.6	0.6	1.8	2.4	1.4
55	1.0	0.4	2.2	2.8	1.7	0.8	0.6	2.0	2.6	1.6
60	1.3	0.6	2.5	3.1	1.9	1.0	0.8	2.4	2.9	1.8
65	1.6	0.6	2.7	3.3	2.0	1.4	1.0	2.8	3.1	2.0
70	1.8	0.8	3.0	3.5	2.3	1.8	1.2	3.0	3.4	2.2
75	2.4	1.2	3.2	4.0	2.6	2.2	1.6	3.4	3.8	2.6
80	2.6	1.4	3.4	4.1	2.8	2.4	2.0	3.8	4.3	2.8
85	3.0	2.2	3.8	4.4	3.1	2.8	2.4	4.2	4.5	3.2
90	3.6	2.7	4.4	5.0	3.6	3.6	3.0	4.6	5.0	3.7
95	5.4	3.3	5.2	5.3	4.3	4.2	3.8	5.2	5.5	4.5
99	6.0	5.9	6.0	6.0	5.7	5.6	5.1	5.9	6.0	5.4

*M* Mean, *SD* Standard deviation

**Table 4:** Comparisons of any and regular occurrences of disordered eating behaviors between gender-expansive participants assigned male sex at birth (AMAB) and those assigned female sex at birth (AFAB)

	Any occurrence (%)				Regular occurrence (%)			
	AMAB (*n* = 135)	AFAB (*n* = 838)	*p*	OR_AMAB-AFAB_	AMAB (*n* = 135)	AFAB (*n* = 838)	*p*	OR_AMAB-AFAB_
	%	%		(95% CI)	%	%		(95% CI)
Dietary Restraint^a^	25.9	22.1	.321	1.24 (0.81, 1.88)	11.1	6.3	.043	1.85 (1.00, 3.40)
Objective binge episodes^a^	17.0	12.2	.117	1.48 (0.90, 2.43)	9.6	6.3	.156	1.58 (0.84, 2.98)
Self-induced vomiting^b^	1.5	1.2	.677	1.25 (0.27, 5.75)	1.5	0.6	.252	2.51 (0.48, 13.05)
Laxative misuse^b^	1.5	1.0	.637	1.56 (0.38, 7.43)	1.5	0.5	.197	3.14 (0.57, 17.29)
Excessive exercise ^a^/^b^	10.4	6.7	.124	1.62 (0.87, 2.99)	3.0	1.0	.072	3.17 (0.94, 10.67)

Any occurrence was defined as ≥1 episode in the past 28 days. Regular occurrence of dietary restraint was defined as going for long periods of time (≥8 h) without eating anything to influence shape or weight for ≥13 days over the past 28 days. Regular occurrence of excessive exercise was defined as exercising in a driven or compulsive way as a means of controlling weight, shape or amount of fat, or burning off calories for ≥20 days over the past 28 days. For all other behaviors (objective binge episodes, self-induced vomiting, and laxative misuse), regular occurrence was defined as ≥4 occurrences over the past 28 days. Definitions for any and regular occurrence are consistent with previously published EDE-Q norms studies [20–22, 24, 25, 28–30]

^a^ Chi-square test

^b^ Fisher’s exact test

Comparisons of eating attitudes and disordered eating behaviors in gender-expansive individuals to transgender men and women are shown in Table 5. There were no significant differences in eating attitudes or disordered eating behaviors noted between gender-expansive individuals and transgender men. Transgender women reported higher Restraint and Shape Concern subscale scores compared to gender-expansive individuals, but there were no significant differences in other eating attitudes or disordered eating behaviors in the two groups.

**Table 5:** Comparisons of eating attitudes and disordered eating behaviors in gender-expansive individuals (*N* = 988) to transgender men (*N* = 312) and transgender women (*N* = 172) from Nagata et al. (2020) [20] in The PRIDE Study

	Gender-expansive individuals from The PRIDE Study	Transgender men from The PRIDE Study [20]			Gender-expansive individuals from The PRIDE Study	Transgender women from The PRIDE Study [20]		
**Eating Attitudes**	Mean (standard deviation)		T-test	*p*	Mean (standard deviation)		T-test	*p*
EDE-Q Restraint	1.24 (1.49)	1.33 (1.42)	−0.98	.326	1.24 (1.49)	1.75 (1.62)	−3.87	< .001*
EDE-Q EC	1.02 (1.26)	0.87 (1.19)	1.85	.065	1.02 (1.26)	0.87 (1.12)	1.38	.168
EDE-Q WC	2.18 (1.63)	2.06 (1.61)	1.20	.230	2.18 (1.63)	2.27 (1.63)	−0.63	.526
EDE-Q SC	2.58 (1.65)	2.65 (1.61)	−0.62	.539	2.58 (1.65)	3.00 (1.68)	−3.09	.002*
EDE-Q Global	1.76 (1.33)	1.73 (1.28)	0.34	.732	1.76 (1.33)	1.98 (1.33)	−1.99	.047
**Disordered eating behaviors**	Any occurrence (%)		Z-test	*p*	Any occurrence (%)		Z-test	*p*
Dietary restraint	23.0	25.5	0.97	.334	23.0	27.9	1.40	.161
Objective binge episodes	12.9	11.2	0.76	.446	12.9	12.8	0.02	.982
Self-induced vomiting	1.4	1.6	^a^	.789	1.4	1.7	^a^	.729
Laxative misuse	1.2	0.3	^a^	.322	1.2	0.6	^a^	.730
Excessive exercise	7.4	8.0	0.36	.716	7.4	8.1	0.35	.730

Any occurrence was defined as ≥1 episode in the past 28 days. EDE-Q scores were compared using independent samples t-tests. Proportions of disordered eating behaviors were compared with Z-tests or Fisher’s exact tests

*EDE-Q* Eating Disorder Examination-Questionnaire, *EDE-Q EC* Eating Concern subscale, *EDE-Q WC* Weight Concern subscale, *EDE-Q SC* Shape Concern subscale, *EDE-Q Global* Global score

* *p* < .005 (after Bonferroni correction)

^a^ Fisher’s exact test

Comparisons from an age-matched (18–26 years) subset of gender-expansive individuals from the current study (*N* = 483) and presumed cisgender men from Lavender et al. [21] are presented in Table 6. Gender-expansive individuals had higher Eating, Weight, and Shape Concern subscales and Global Score compared to presumed cisgender men. However, gender-expansive individuals reported a lower frequency of objective binge episodes and excessive exercise compared to presumed cisgender men. Comparisons from an age-matched (18–25 years) subset of gender-expansive individuals from the current study (*N* = 434) and presumed cisgender women from Luce et al. [22] are also presented in Table 6. Age-matched gender-expansive participants showed significantly lower scores on the Restraint subscale, but higher scores on the Shape Concern subscale than presumed cisgender women. In terms of disordered eating behaviors, gender-expansive participants from the current study showed significantly lower frequencies of self-induced vomiting, laxative misuse, and excessive exercise than presumed cisgender women.

**Table 6:** Comparisons of eating attitudes and disordered eating behaviors in a subsample of gender-expansive individuals 18–26 years old (*N* = 483) and 18–25 years old (*N* = 434) in The PRIDE Study to age-matched cisgender^a^ men from the Lavender et al. (2010) [21] sample (*N* = 404) and cisgender^a^ women from the Luce et al. (2008) [22] sample (*N* = 723)

	Gender-expansiveindividuals fromThe PRIDE Study 18–26 years	Cisgender^a^ menfrom Lavenderet al. (2010) [21]			Gender-expansiveindividuals fromThe PRIDE Study 18–25 years	Cisgender^a^ womenfrom Luce et al.(2008) [22]		
**Eating Attitudes**	Mean (standard deviation)		T-test	*p*	Mean (standard deviation)		T-test	*p*
EDE-Q Restraint	1.15 (1.46)	1.04 (1.19)	1.21	.227	1.15 (1.45)	1.62 (1.54)	−5.14	< .001*
EDE-Q EC	1.07 (1.27)	0.43 (0.77)	9.86	< .001*	1.10 (1.27)	1.11 (1.11)	−0.14	.889
EDE-Q WC	2.18 (1.66)	1.29 (1.27)	8.83	< .001*	2.20 (1.66)	1.97 (1.56)	2.37	.018
EDE-Q SC	2.56 (1.68)	1.59 (1.38)	9.23	< .001*	2.59 (1.69)	2.27 (1.54)	3.30	< .001*
EDE-Q Global	1.74 (1.36)	1.09 (1.00)	7.97	< .001*	1.76 (1.36)	1.74 (1.30)	0.25	.803
**Disordered eating behaviors**	Any occurrence (%)		Z-test	*p*	Any occurrence (%)		Z-test	*p*
Dietary restraint	27.1	24.0	0.80	.423	27.4	25.9	0.581	.562
Objective binge episodes	14.3	25.0	4.04	< .001*	15.0	21.3	2.66	.008
Self-induced vomiting	1.4	3.2	1.77	.077	1.4	8.8	5.16	< .001*
Laxative misuse	1.2	2.7	1.60	.109	1.2	8.3	5.11	< .001*
Excessive exercise	8.1	31.4	8.88	< .001*	8.1	30.8	9.01	< .001*

Any occurrence was defined as ≥1 episode in the past 28 days. EDE-Q scores were compared using independent samples t-tests. Proportions of disordered eating behaviors were compared with Z-tests or Fisher’s exact tests

*EDE-Q* Eating Disorder Examination-Questionnaire, *EDE-Q EC* Eating Concern subscale, *EDE-Q WC* Weight Concern subscale, *EDE-Q SC* Shape Concern subscale, *EDE-Q Global* Global score

* *p* < .005 (after Bonferroni correction)

^a^ Cisgender is presumed here as comprehensive gender assessment was not performed in Lavender et al. (2010) [21] or Luce et al. (2008) [22]

## Discussion

In this study, we detail the community norms for the EDE-Q among gender-expansive individuals. Developing this understanding is important as the population of gender-expansive individuals is highly understudied, and their health outcomes have seldom been studied as distinct from other gender minorities [33]. Although it has been previously proposed that all gender minority people may be at an elevated risk of eating pathology [6, 8], the gender-expansive population faces distinct psychosocial stressors, which may contribute to maladaptive eating attitudes and behaviors [1, 34]. Understanding the manifestations of eating disorders in this population is important for developing targeted risk assessments and treatment approaches.

Compared to transgender men in The PRIDE study, the EDE-Q subscale and global scores in this population were similar. However, transgender women had higher Restraint and Shape Concern subscale scores than gender-expansive individuals. This may be due to gender-expansive and transgender individuals experiencing eating disorders as a result of discrimination, stigma, and prejudice that they encounter secondary to their gender identity, termed the Gender Minority Stress Theory [15, 34]. Specifically, gender-expansive individuals may face psychological distress due to identity invalidation, decreased social support, and increased discrimination [1, 2, 17]. However, transgender men and women may be differentially impacted by gender-specific body ideals [20]. Overall, it is important for clinicians to recognize that gender minority groups face distinct stressors that elevate their risk of disordered eating attitudes and behaviors.

Presumed cisgender women had higher Restraint subscale scores and presumed cisgender men had lower Global scores and Eating Concern, Weight Concern, and Shape Concern subscale scores compared to gender-expansive individuals. These differences may reflect societal gender norms, with greater pressures for thinness in women than men [35, 36]. Gender-expansive individuals reported significantly higher scores on the Shape Concern subscale than presumed cisgender women. Similarly, transgender women previously reported higher scores on the Shape Concern subscale than presumed cisgender women [20]. Shape concerns in gender-expansive individuals may be linked to gender dysphoria and body dissatisfaction when one’s body shape is discrepant from one’s gender identity [18, 37, 38]. It may be especially challenging for gender-expansive individuals to attain certain shape ideals when certain anatomic structures cannot be altered medically or surgically [38, 39].

Among this population of gender-expansive participants, nearly a quarter of participants reported engaging in dietary restraint at least once in the previous 28 days, and 7.4% reported doing so for about half or more of the previous 28 days. Additionally, 12.9% reported engaging in at least one objective binge episode, with about half of them reporting regular binge episodes. Similar to transgender men and women, this population reported elevated rates of all disordered eating behaviors [20, 26]. Presumed cisgender women reported more frequent vomiting and laxative use than gender-expansive individuals, which may reflect gender norms and society pressures for thinness in women [35]. Presumed cisgender men had a higher frequency of excessive exercise than gender-expansive individuals, which may reflect gender norms and greater societal pressures for muscularity in men [35]. Presumed cisgender men had a higher frequency of objective binge episodes than gender-expansive individuals. Given higher Eating, Weight, and Shape Concern subscale scores, gender-expansive individuals may attempt to avoid binge eating more than cisgender men. Among cisgender men, binge eating may be termed “cheat meals” and deemed positive in the context of muscularity-oriented goals [40].

Interestingly, we found that there were no statistically significant differences in reported behavior occurrences between gender-expansive individuals AMAB and AFAB. This contrasts with well-established differences between cisgender men and women in terms of ED prevalence and symptomology [41, 42]. Transgender men and women report different manifestations of eating disorder attitudes and behaviors [43]. Our findings suggest that, unlike in cisgender and binary gender-identified transgender individuals [20], sex assigned at birth is not associated with risk differences or able to anticipate manifestations of eating psychopathology among gender-expansive individuals. Furthermore, this suggests that gender-expansive individuals may be less impacted by binary gender-specific body ideals, which have been implicated in the differences seen in both cisgender and transgender populations [12, 41, 43]. Overall, this further affirms the importance of understanding a patient through the lens of their own gender identity without trying to fit non-binary individuals into a binary medical understanding of the disorder, a concern that has been cited by non-binary patients [44].

There are a few important limitations of the present study. First, the participants of The PRIDE study are predominantly non-Latino, White, and highly educated. As research suggests that nearly one third of gender-expansive people are not White, these populations are underrepresented in the present study [45]. Overall, this may limit the generalizability of these results to people of color. Additionally, as the mechanism of recruitment was primarily online and the participants were self-selected, there is additional consideration warranted for the external validity of the results that should be taken into account. A second limitation to consider is that the gender-expansive community is heterogeneous, but, for this paper, all gender identities other than exclusively cisgender or binary transgender were combined into one group. As a result, potential differences between specific gender-expansive identities *(*e.g., agender versus genderqueer) may be obscured. Finally, there are limitations with the comparison groups used, including different recruitment methods (college students who were participating as part of course requirements), the potential effects of time on responses (given that the comparison groups were recruited approximately 10 years prior), and the comparison of only a subset of young adults as opposed to our entire sample [21, 22].

## Conclusions

We present for the first time norms of the EDE-Q among gender-expansive individuals, which can aid clinicians and researchers in interpreting EDE-Q scores in this understudied population. The present results suggest several next steps for future research on eating pathology in gender-expansive individuals. Specifically, it will be important to apply these results to actionable clinical suggestions that can help guide providers who care for gender-expansive individuals. Clinicians caring for gender-expansive individuals should assess for their current body ideals and how they modify their behaviors to achieve them. Additionally, it will be important to understand if there are key differences between specific identities within the gender-expansive population.

## Data Availability

Data from The PRIDE Study may be accessed through an Ancillary Study application (details at pridestudy.org/collaborate).

## Appendix

**Table 7:** Population definitions excluded from gender-expansive classification

Population	Sex assigned at birth	Gender identity
Cisgender man	male	man
Cisgender woman	female	woman
Transgender man	female	man, transgender man, or transmasculine
Transgender woman	male	woman, transgender woman, or transfeminine

## Abbreviations

AFAB: Assigned female sex at birth
AMAB: Assigned male sex at birth
BMI: Body mass index
EC: Eating Concern subscale
ED: Eating disorder
EDE-Q: Eating Disorder Examination Questionnaire
M: Mean
PRIDE Study: Population Research in Identities and Disparities for Equality (PRIDE) Study
R: Restraint subscale
SC: Shape Concern subscale
SD: Standard deviation
WC: Weight Concern subscale
